# Side‐On Bonded Beryllium Dinitrogen Complexes

**DOI:** 10.1002/anie.202002621

**Published:** 2020-04-14

**Authors:** Guohai Deng, Sudip Pan, Guanjun Wang, Lili Zhao, Mingfei Zhou, Gernot Frenking

**Affiliations:** ^1^ Collaborative Innovation Center of Chemistry for Energy Materials Department of Chemistry Shanghai Key Laboratory of Molecular Catalysts and Innovative Materials Fudan University Shanghai 200438 China; ^2^ Institute of Advanced Synthesis School of Chemistry and Molecular Engineering Jiangsu National Synergetic Innovation Center for Advanced Materials Nanjing Tech University Nanjing 211816 China; ^3^ Fachbereich Chemie Philipps-Universität Marburg Hans-Meerwein-Strasse 4 35043 Marburg Germany

**Keywords:** beryllium, nitrogen, IR spectroscopy, matrix isolation, structure elucidation

## Abstract

The preparation and spectroscopic identification of the complexes NNBe(η^2^‐N_2_) and (NN)_2_Be(η^2^‐N_2_) and the energetically higher lying isomers Be(NN)_2_ and Be(NN)_3_ are reported. NNBe(η^2^‐N_2_) and (NN)_2_Be(η^2^‐N_2_) are the first examples of covalently side‐on bonded N_2_ adducts of a main‐group element. The analysis of the electronic structure using modern methods of quantum chemistry suggests that NNBe(η^2^‐N_2_) and (NN)_2_Be(η^2^‐N_2_) should be classified as π complexes rather than metalladiazirines.

## Graphical abstract

**The good side**: The preparation and spectroscopic identification of the complexes NNBe(η^2^‐N_2_) and (NN)_2_Be(η^2^‐N_2_) and the energetically higher lying isomers Be(NN)_2_ and Be(NN)_3_ are reported. NNBe(η^2^‐N_2_) and (NN)_2_Be(η^2^‐N_2_) are the first examples of covalently side‐on bonded N_2_ adducts of a main‐group element. The analysis of the electronic structure using modern methods of quantum chemistry suggests that NNBe(η^2^‐N_2_) and (NN)_2_Be(η^2^‐N_2_) should be classified as π complexes rather than metalladiazirines.

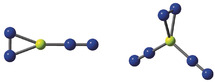

## Introduction

**Figure 1:**
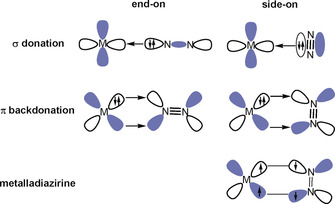
Schematic diagram of the bonding interactions between a transition metal and dinitrogen in the end‐on‐(left) and side‐on (right) bonded metal dinitrogen complexes.

Dinitrogen activation and its chemical transformations are one of the most challenging subjects in chemistry. Coordination of N_2_ to transition‐metal centers is an effective strategy for activation. Many transition metals have been found to bind N_2_ following the Dewar–Chatt–Duncanson (DCD) bonding model,^1^ which involves synergic σ donation from N_2_ into an empty orbital of a metal and π back‐donation of electron density from the occupied metal d orbitals into the antibonding π orbitals of N_2_ (Figure 1).^2^, ^3^, ^4^, ^5^, ^6^ The π back‐donation weakens the strong N–N triple bonds, which is crucial for its further activation, its complete N−N bond cleavage, and functionalization. Dinitrogen can bind with transition metal and actinide metal centers in various modes. The manner by which N_2_ binds to the metal centers is a key factor to influence the extent of activation and subsequent functionalization propensity.^7^, ^8^ The terminal end‐on coordination is the most prevalent binding mode observed for N_2_, in which the HOMO of N_2_ serves as the donating orbital (Figure 1). The side‐on bonding is yet another binding mode to a mononuclear metal center. In this mode, the π‐bonding orbital of N_2_ (HOMO‐1) acts as the donor orbital (Figure 1). As both σ donation and π back‐donation weaken the N−N bond, the side‐on mode results in a more activated N_2_ ligand than the end‐on coordination. However, this mode is very uncommon as the HOMO‐1 π‐bonding orbital of N_2_ lies lower in energy than the HOMO.^9^ A similar binding mode is also operative in dinitrogen complexes of the f‐block elements, which exhibit a rich chemistry particularly for uranium.^10^

Generally, main‐group elements are reluctant to bind N_2_ in forming stable complexes. Only very few weakly coordinated dinitrogen complexes of main‐group elements have been observed in low‐temperature noble‐gas matrices or in the gas phase.^6^, ^11^, ^12^, ^13^, ^14^, ^15^, ^16^, ^17^, ^18^, ^19^, ^20^ The main‐group complexes are predominantly end‐on‐bound, with the exception of the LiN_2_ complex, for which a side‐bound structure with almost purely ionic bonding has been proposed.^20^ Recent studies show that reactive borylenes can bind N_2_ to form stable dinitrogen adducts and the reductive coupling of two hypovalent‐boron‐bound N_2_ units mimics transition metals, which can be further protonated to derivatives,^21^, ^22^ demonstrating the feasibility of nitrogen fixation and reduction by nonmetal main‐group species.^23^ Here we report a joint matrix‐isolation infrared spectroscopic and theoretical study on beryllium dinitrogen complexes involving a highly activated side‐on bonded dinitrogen ligand.

Beryllium is by far the least‐investigated naturally occurring non‐radioactive element in the periodic table as it has long been regarded as the most toxic non‐radioactive element.^24^ Recent experimental and theoretical studies have provided evidence for the rich and versatile chemistry of beryllium.^25^, ^26^, ^27^, ^28^, ^29^, ^30^ Owing to its relatively high electronegativity and small atomic radius, the beryllium atom has the tendency to form covalent bonds with other elements, unlike the heavier alkaline‐earth metals. The structure, bonding, and reactivity of simple beryllium compounds serve as excellent model systems in achieving a thorough understanding of beryllium chemistry.

## Results and Discussion

### Experimental Studies

**Figure 2:**
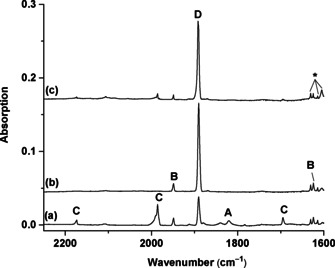
Infrared spectra in the 2250–1600 cm^−1^ region from co‐deposition of laser‐evaporated beryllium atoms with 0.5 % N_2_ in neon. a) After 30 min of sample deposition at 4 K. b) After 4 min of 617 nm light irradiation. and c) After annealing to 10 K. **A**: Be(NN)_2_; **B**: NNBe(η^2^‐N_2_); **C**: (NN)_2_Be(η^2^‐N_2_); **D**: Be(NN)_3_. The * denotes water absorptions.

The beryllium dinitrogen complexes were prepared by the reactions of pulsed laser evaporated beryllium atoms and dinitrogen in solid neon at 4 K. The product species were detected using infrared absorption spectroscopy.^31^ The experiments were performed using relatively low laser energy to avoid the formation of multinuclear species. The previously reported dinuclear species are barely observed.^11^ Figure 2 shows the spectra in the N–N stretching frequency region from the experiment using a 0.5 % N_2_/Ne sample (on the basis of volume). The spectra in the Be–N stretching region are shown in Figure S1 (see the Supporting Information). Several new product absorptions are observed, and can be classified into four groups based on their annealing and photochemical behaviors (labeled as **A**–**D** in Figure 2). Only one band is observed for species **A**, which is observed on sample deposition and is destroyed upon irradiation with 617 nm light. The species **B** involves three absorptions with comparable intensities (Table 1), which appear on sample deposition, remain unchanged upon irradiation with 617 nm light. The group **C** absorptions appear on sample deposition and are completely destroyed under irradiation with 617 nm light, during which the group **D** absorptions markedly increase. All these absorptions remain almost unchanged or slightly decrease upon sample annealing when the matrix is annealed first and then irradiated. All of them remain unchanged under irradiation from the source of the IR spectrometer, but are completely destroyed under yellow (580–595 nm), blue (435–480 nm), and UV (*λ*>250 nm) light. Experiments with isotopically substituted N_2_ samples allow the unambiguous identification of these absorptions through isotopic shifts and splittings (see Figures S2–S5). An experiment using pure dinitrogen as a matrix was also performed (see Figure S6). Only the absorptions of **C** and **D** are observed in solid nitrogen, suggesting that both species are saturate‐coordinated complexes. The species **C** is converted into **D** under irradiation with 617 nm light as observed in solid neon.

**Table 1:** Experimental (Ne) and (scaled) calculated vibrational frequencies (cm^−1^) and isotopic shifts Δ (cm^−1^) of the beryllium dinitrogen complexes.

Species	Exptl^[a]^			Calcd^[b,c,d]^		
	^14^N_2_	^15^N_2_	Δ	^14^N_2_	^15^N_2_	Δ
Be(NN)_2_ (**A**)	1820.4	1763.5	−56.9	1942.5 (3742)	1878.3	−64.2
NNBe(*η* ^2^‐N_2_) (**B**)	1948.9(1.00)	1887.6	−61.3	2003.0 (863)	1935.6	−67.4
	1624.1(0.67)	1577.8	−46.3	1621.9 (674)	1572.9	−49.0
	1030.2(0.50)	1022.5	−7.7	1072.4 (926)	1063.7	−8.7
(NN)_2_Be(*η* ^2^‐N_2_) (**C**)	2173.5(0.11)	2101.5	−72.0	2207.0 (329)	2132.2	−74.8
	1985.8(1.00)	1920.7	−65.1	2045.7 (1297)	1976.7	−69.0
	1694.9(0.17)	1647.0	−47.9	1688.9 (1045)	1633.8	−55.1
	819.0(0.06)	813.3	−5.7	848.3 (399)	842.1	−6.2
	622.8(0.11)	619.5	−3.3	672.6 (64)	668.1	−4.5
Be(NN)_3_ (**D**)	1890.3(1.00)	1828.8	−61.5	1942.0 (2098)	1876.4	−65.6
	836.5/825.0(0.10)	831.1/819.8	−5.4/−5.2	877.8 (131)	871.2	−6.6

[a] The values within parentheses are the integrated intensities normalized to the most intense absorption. [b] The calculated IR intensities are listed in parentheses in km mol^−1^ at the M06‐2X‐D3/cc‐pVTZ level. [c] The calculated values refer to isomers **1**(**B**), **2**(**A**), **5**(**C**) and **6**(**D**). [d] The calculated harmonic frequencies are scaled with the factor 0.9848, which is obtained from the ratio of experimental stretching frequency of 2330 cm^−1^ for N_2_ and the calculated value of 2366 cm^−1^.

The species **A**, with absorption at 1820.4 cm^−1^ in Ne, can be identified as the Be(NN)_2_ complex with two end‐on bound N_2_ ligands on the basis of spectra in the experiments with 0.25 % N_2_ + 0.25 % ^15^N_2_ and 0.25 % N_2_ + 0.5 % ^14^N^15^N + 0.25 % ^15^N_2_ mixed samples (see Figure S2). The band position is only 3.0 cm^−1^ red‐shifted from the argon matrix value.^11^ The species **B** is assigned to the NNBe(η^2^‐N_2_) isomer involving one end‐on and one side‐on bonded N_2_ ligands. The 1948.9 cm^−1^ band is the N–N stretching mode of the end‐on bonded N_2_ ligand, while the 1624.1 cm^−1^ band is the N–N stretching mode of the side‐on bound ligand with two equivalent N atoms. The 1030.2 cm^−1^ band originates from the Be–N stretching vibration. The group **C** absorptions are assigned to different vibrational modes of the (NN)_2_Be(η^2^‐N_2_) complex (Table 1). The 2173.5 and 1985.8 cm^−1^ bands are symmetric and antisymmetric, respectively, N–N stretching modes of the two equivalent end‐on bound N_2_ ligands. The 1694.9 cm^−1^ band is the N–N stretching vibration of the side‐on bonded N_2_ ligand. The 819.0 cm^−1^ band is attributed to the Be‐N_2_ stretching mode, while the 622.8 cm^−1^ band is due to the antisymmetric NN‐Be‐NN stretching mode. These modes are observed at 2183.6, 2007.1, 1683.0, 820.5, and 622.3 cm^−1^ in solid nitrogen. The species **D** is assigned to the Be(NN)_3_ complex. Only one band at 1890.3 cm^−1^ is observed in the N–N stretching frequency region in Ne, and it is due to the doubly degenerate N–N stretching mode. The isotopic splitting patterns in the experiments with 0.25 % N_2_ + 0.25 % ^15^N_2_ and 0.25 % N_2_ + 0.5 % ^14^N^15^N + 0.25 % ^15^N_2_ samples (see Figure S2) confirm that three equivalent N_2_ ligands are involved in this mode. The 836.5 and 825.0 cm^−1^ bands are attributed to the B–N stretching mode in two trapping sites. These two modes are observed at 1890.0 and 843.6/817.5 cm^−1^ in solid nitrogen.

### Theoretical Studies

**Figure 3:**
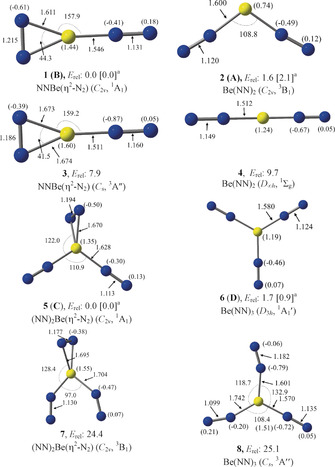
Optimized equilibrium geometries of Be(NN)_2_ **1**–**4** and Be(NN)_3_ **5**, **6** at the CCSD(T)‐Full/cc‐pVTZ level. Be(NN)_3_ **7**, **8** are studied at the CCSD(T)‐Full/cc‐pVTZ//CCSD/cc‐pVTZ level. Relative energies are given in kcal mol^−1^. Bond distances are given in Å and angles in degree. The NBO atomic partial charges are given in parentheses. ^a^Values in square brackets are calculated at the CCSD(T)‐Full/aug‐cc‐pVQZ//CCSD(T)‐Full/cc‐pVTZ level.

We calculated the structures and properties of isomeric forms of Be(NN)_*n*_, where *n*=2 or 3, using ab initio methods at the CCSD(T)‐Full/cc‐pVTZ level and using density‐functional theory (DFT) at the M06‐2X‐D3/cc‐pVTZ level (see Figure S7 for DFT results). Details of the methods are given in the Supporting Information. Figure 3 shows the optimized geometries and the calculated bond lengths and angles of the equilibrium structures in the electronic singlet and triplet states. The calculations suggest that the most stable form of Be(NN)_2_ is the NNBe(η^2^‐N_2_) isomer **1** with *C*_2*v*_ symmetry in the singlet (^1^A_1_) electronic state. The double end‐on isomer **2** has a triplet (^3^B_1_) electronic state and a bent *C*_2*v*_ geometry with a bending angle of 108.8°. The structure **2** is only 1.6 kcal mol^−1^ higher in energy than **1**. The energy difference is slightly larger (2.1 kcal mol^−1^) when a larger basis set at the CCSD(T)‐Full/aug‐cc‐pVQZ level is employed. Energetically, the next low‐lying isomer, **3**, in the triplet (^3^A′′) electronic state with *C*_s_ symmetry is 7.9 kcal mol^−1^ above **1** (Figure 3). The linear Be(NN)_2_ species **4** in the electronic singlet (^1^Σ_g_) state is 9.7 kcal mol^−1^ higher lying than **1**. Two energetically close isomers, **5** and **6**, in the electronic singlet state are predicted as the most stable forms of Be(NN)_3_. The (NN)_2_Be(η^2^‐N_2_) isomer **5** with *C*_2*v*_ symmetry is only 1.7 kcal mol^−1^ (0.9 kcal mol^−1^ at CCSD(T)‐Full/aug‐cc‐pVQZ) lower in energy than the *D*_3*h*_ symmetric structure **6** of Be(NN)_3_. The triplet isomers (NN)_2_Be(η^2^‐N_2_) **7** and Be(NN)_3_ **8** are 24.4 and 25.1 kcal mol^−1^, respectively, higher in energy than **5** at the CCSD(T)‐Full/cc‐pVTZ//CCSD/cc‐pVTZ level.

The calculations at the CCSD(T)‐Full/aug‐cc‐pVQZ level thus predict that the cyclic species with side‐on bonded η^2^‐N_2_ ligands, **1** and **5**, are the energetically lowest lying isomers of Be(NN)_2_ and Be(NN)_3_, respectively, with the end‐on bonded forms **2** and **6** being slightly higher in energy. We assigned the spectroscopically identified species **A**—**D** to the four energetically lowest lying isomers as **1**(**B**), **2**(**A**), **5**(**C**), and **6**(**D**). Table 1 shows the calculated vibrational frequencies of **1**, **2**, **5**, and **6** in the experimentally recorded range. The full set of calculated frequencies is given in Tables S2 and S3. The calculated harmonic frequencies are scaled with the factor 0.9848, which is obtained from the ratio of experimental stretching frequency of 2330 cm^−1^ for N_2_ 32 and the calculated value of 2366 cm^−1^. The agreement between the calculated harmonic frequencies and the anharmonic experimental values is quite good. This agreement is particularly good for the N–N stretching mode of the side‐on bonded N_2_ ligands in **1**(**B**) and **5**(**C**), where the theoretical and the observed frequencies nicely match. Further support for the assignment of the vibrational spectra of **A**–**D** to the four energetically lowest lying isomers **1**, **2**, **5**, and **6** comes from the ^14^N/^15^N isotope shifts Δ, which span a range between −3 and −75 cm^−1^. The nearly perfect agreement between the calculated and experimental isotope shifts leaves no doubt that the assignments **1**(**B**), **2**(**A**), **5**(**C**), and **6**(**D**) are correct.

We analyzed the nature of the beryllium–dinitrogen interactions with charge‐ and energy‐partitioning methods. Figure 3 shows also the atomic partial charges in the calculated species. As expected, the beryllium atom carries a large positive charge while the dinitrogen ligands are negatively charged. The positive charge at Be in the side‐on species is always somewhat larger than in the other isomers. The question arises if the species with a side‐on bonded η^2^‐N_2_ ligand are better described in terms of donor–acceptor interactions following the DCD model,^1^ or as metalladiazirines, which are cyclic compounds with genuine Be–N electron sharing bonds. Both models are schematically shown in Figure 1. The dichotomy of the two bonding models is well known in transition‐metal chemistry, where, for example, π complexes of alkenes and alkynes are distinguished from metallacyclopropanes and metallacyclopropenes.^33^ Diazirines (R_2_CN_2_) are experimentally studied compounds and the structures of the parent systems with R=H, F, and CH_3_, are available from the gas phase measurements.^34^ The question as to whether **1** and **5** should be considered metalladiazirines or π‐bonded η^2^‐N_2_ complexes can be addressed with energy decomposition analysis (EDA) in combination with natural orbital for chemical valence (NOCV) theory using N_2_ and Be(NN)_*n*_ (*n*=1,2) in the electronic triplet and singlet states as interacting species. A particular strength of the EDA‐NOCV method lies in its ability to determine the most suitable fragments for the formation of a chemical bond, which is given by the smallest change in the associated orbital interaction, Δ*E*_orb_. This ability has been demonstrated for a variety of chemical bonds.^35^

**Figure 4:**
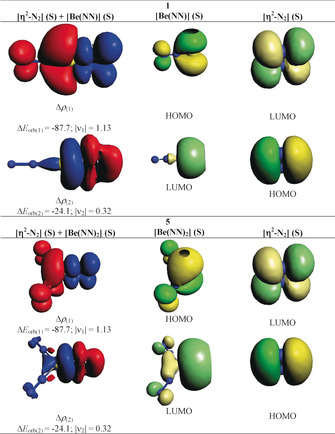
Shape of the deformation densities, Δ*ρ*_(1)‐(2)_, of **1** and **5** corresponding to Δ*E*_orb(1)_ and Δ*E*_orb(2)_, respectively, and the associated fragment orbitals at the M06‐2X/TZ2P level. Isosurface values are 0.001 au. The eigenvalues |ν_n_| give the size of the charge migration in e. The direction of the charge flow of the deformation densities is red→blue.

Table 2 shows the numerical results of the EDA‐NOCV results of **1** and **5**. The calculations for both molecules give much smaller Δ*E*_orb_ values when singlet fragments are employed instead of triplet fragments. These values mean that the fragments in the electronic singlet states undergo much less change in the electronic structure and thus, are better prepared for bond formation than the triplet fragments. The EDA‐NOCV results suggest that both **1** and **5** should be classified as π‐bonded η^2^‐N_2_ complexes rather than metalladiazirines. Figure 4 shows the deformation densities, Δ*ρ*_(1)‐(2)_, of **1** and **5** that are associated with Δ*E*_orb(1)_ and Δ*E*_orb(2)_, and the interacting donor and acceptor fragment orbitals. It becomes obvious that the strongest interaction, Δ*E*_orb(1)_, comes from (NN)_*n*_Be→η^2^‐N_2_ π back‐donation while the much weaker second strongest orbital term, Δ*E*_orb(2)_, is due to (NN)_*n*_Be←η^2^‐N_2_ σ donation. The dominant orbital interactions in **1** and **5** are archetypical examples of HOMO/LUMO donation and back‐donation following the frontier orbital theory of Fukui,^36^ where the vacant in‐plane π_∥_* orbital of N_2_ serves as an acceptor MO in the stronger orbital interaction, Δ*E*_orb(1)_, and the occupied in‐plane π_∥_ orbital of N_2_ is the donor part in the weaker orbital interaction Δ*E*_orb(2)_.

**Table 2:** EDA‐NOCV results of **1** and **5** at the M06‐2X/TZ2P//CCSD(T)‐Full/cc‐pVTZ level taking η^2^‐N_2_ and Be(NN)_*n*_ in the singlet or triplet states as interacting fragments. Energy values are in kcal mol^−1^.

Energy terms	**1**		**5**	
	[η^2^‐N_2_] (S) + [Be(NN)] (S)	[η^2^‐N_2_] (T) + [Be(NN)] (T)	[η^2^‐N_2_] (S) + [Be(NN)_2_] (S)	[η^2^‐N_2_] (T) + [Be(NN)_2_] (T)
Δ*E*_int_	−100.6	−195.4	−64.3	−188.8
Δ*E*_Pauli_	96.6	291.6	77.3	213.4
Δ*E*_Metahybrid_	4.4	6.3	0.2	10.2
Δ*E*_elstat_^[a]^	−27.0 (13.4 %)	−184.1 (37.3 %)	−20.0 (14.1 %)	−116.8 (28.3 %)
Δ*E*_orb_^[a]^	−174.6 (86.6 %)	−309.2 (62.7 %)	−121.8 (85.9 %)	−295.5 (71.7 %)
Δ*E*_orb(1)_^[b]^	−135.0 (77.3 %)	−229.3 (74.2 %)	−87.7 (72.0 %)	−242.7 (82.1 %)
Δ*E*_orb(2)_^[b]^	−24.0 (13.7 %)	−54.3 (17.6 %)	−24.1 (19.8 %)	−43.3 (14.7 %)
Δ*E*_orb(rest)_^[b]^	−15.6 (8.9 %)	−25.6 (8.3 %)	−10.0 (8.2 %)	−9.5 (3.2 %)

[a] The values within the parentheses show the contribution to the total attractive interaction Δ*E*_elstat_ + Δ*E*_orb_. [b] The values within parentheses show the contribution to the total orbital interaction Δ*E*_orb_.

**Figure 5:**
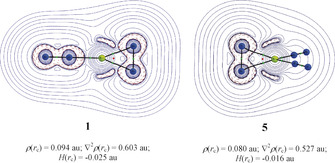
The contour plot of the Laplacian of electron density, ∇^2^ *ρ*(*r*_c_), in the plane of Be(η^2^‐N_2_) in **1** and **5**. The values of topological descriptors are provided for the BCP between Be and η^2^‐N_2_. The blue solid lines indicate regions of charge depletion (∇^2^ *ρ*(*r*_c_)>0) and red dotted lines indicate regions of charge accumulation (∇^2^ *ρ*(*r*_c_)<0). Green and red spheres represent bond critical points and ring critical points, respectively. The calculations were carried out at CCSD‐Full/cc‐pVTZ using the CCSD(T)‐Full/cc‐pVTZ optimized structures.

The results of the EDA‐NOCV calculations of **1** and **5** are nicely complemented by the QTAIM analysis of the electronic structures. Figure 5 shows the Laplacian distribution of the two complexes in the plane of the cyclic moiety. There is an area of local charge concentration (∇^2^ *ρ*(*r*_c_)<0; red dashed lines) at the beryllium atoms pointing in the direction toward the nitrogen atoms of the η^2^‐N_2_ ligands. This unusual scenario supports the classification of **1** and **5** as π‐bonded η^2^‐N_2_ complexes where the dominant orbital contribution is due to (NN)_*n*_Be→η^2^‐N_2_ π back‐donation.

**Figure 6:**
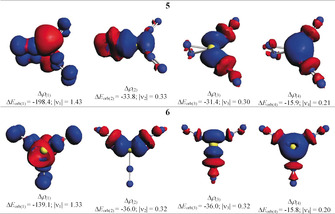
Shape of the deformation densities, Δ*ρ*_(1)‐(4)_, of **5** and **6** corresponding to Δ*E*_orb(1)_–Δ*E*_orb(4)_ at the M06‐2X/TZ2P level. Isosurface values are 0.001 au. The eigenvalues |ν_n_| give the size of the charge migration in e. The direction of the charge flow is red→blue.

We also analyzed the side‐on and end‐on bonded dinitrogen complexes with the EDA‐NOCV method to elucidate the peculiar stability of the former bonding mode. The calculations were restricted to **5** and **6** because they are both in the electronic singlet state. We choose the neutral fragments of Be in the singlet state with the electronic reference configuration (2s^0^2p^2^), and the three dinitrogen ligands in the singlet state as interacting moieties, which refer to the symmetry‐allowed dissociation products. The alignment of the occupied 2p AO of beryllium was chosen following the occupied MOs of **5** and **6**. The numerical results are shown in Table 3. The intrinsic interaction energy between the beryllium atom and the dinitrogen ligands (Δ*E*_int_) in the side‐on bonded (NN)_2_Be(η^2^‐N_2_) complex **5** is larger (−211.8 kcal mol^−1^) than in the end‐on bonded isomer **6** (−199.8 kcal mol^−1^). The inspection of the energy terms indicates that this difference comes mainly from the attractive contributions of the Coulomb term Δ*E*_elstat_ and particularly from the covalent (orbital) interactions (Δ*E*_orb_). The breakdown of the latter term into pairwise orbital interactions reveals that the dominant orbital stabilization, Δ*E*_orb(1)_, is due to the back‐donation of the 2p electron pair of beryllium into the π* MO of the N_2_ ligands. The Δ*E*_orb(1)_ term in **5** is much larger (−198.4 kcal mol^−1^) than in the end‐on bonded isomer **6** (−139.1 kcal mol^−1^). The associated deformation densities Δρ_(1)_ shown in Figure 6 reveal that the π back‐donation Be(2s^0^2p^2^)→η^2^‐N_2_ into the in‐plane π_∥_* orbital of the side‐on bonded ligand is much larger than the back‐donation into the end‐on bonded N_2_ ligands. This difference comes from the larger overlap of the in‐plane π_∥_* orbital of the side‐on bonded N_2_ with the 2p AO of Be. The result has bearings for future studies of dinitrogen complexes. Complexes with side‐on bonded N_2_ ligands may be favored over end‐on bonded isomers when the π back‐donation from the metal to the ligands is particularly strong. Isomers of Be(NN)_*n*_ with more than one side‐on bonded (η^2^‐N_2_) ligands are not minima on the potential energy surface because there is only one electron pair of the metal available for π back‐donation.

**Table 3:** EDA‐NOCV results of **5** and **6** at the M06‐2X/TZ2P//CCSD(T)‐Full/cc‐pVTZ level using Be atom in the singlet state with the electron configuration (2s^0^2p^2^) and the three N_2_ ligands as interacting fragments. Energy values are in kcal mol^−1^.

Energy terms	Orbital interaction	5	6
		[Be] (S, 2s^0^2p_∥_^2^)^[c]^ + [(NN)_2_(η^2^‐N_2_)] (S)	[Be] (S, 2s^0^2p_⊥_^2^)^[d]^ + [(NN)_3_] (S)
Δ*E*_int_		−211.8	−199.8
Δ*E*_Pauli_		159.3	95.4
Δ*E*_Metahybrid_		16.0	24.6
Δ*E*_elstat_^[a]^		−82.4 (21.3 %)	−65.1 (20.4 %)
Δ*E*_orb_^[a]^		−304.8 (78.7 %)	−254.7 (79.6 %)
Δ*E*_orb(1)_^[b]^	[Be] (2p^2^)→[(NN)_2_(η^2^‐N_2_)]/[(NN)_3_] π‐back‐donation	−198.4 (65.1 %)	−139.1 (54.6 %)
Δ*E*_orb(2)_^[b]^	[Be] (2p^0^)←[(NN)_2_(η^2^‐N_2_)]/[(NN)_3_] σ‐donation	−33.8 (11.1 %)	−36.0 (14.1 %)
Δ*E*_orb(3)_^[b]^	[Be] (2p^0^)←[(NN)_2_(η^2^‐N_2_)]/[(NN)_3_] σ‐donation	−31.4 (10.3 %)	−36.0 (14.1 %)
Δ*E*_orb(4)_^[b]^	[Be] (2s^0^)←[(NN)_2_(η^2^‐N_2_)]/[(NN)_3_] σ‐donation	−15.9 (5.2 %)	−15.8 (6.2 %)
Δ*E*_orb(rest)_^[b]^		−46.2 (18.2 %)	−27.8 (10.9 %)

[a] The values within the parentheses show the contribution towards the total attractive interaction Δ*E*_elstat_ + Δ*E*_orb_. [b] The values within the parentheses show the contribution towards the total orbital interaction, Δ*E*_orb_. [c] The occupied 2p_∥_ AO is in the plane of the Be‐(η^2^‐N_2_) moiety. [d] The occupied 2p_**⊥**_ AO is in the molecular plane.

The EDA‐NOCV results in Table 3 suggest that the beryllium‐dinitrogen bonds in **5** and **6** have a stronger covalent than electrostatic character. The orbital term Δ*E*_orb_ accounts for almost 80 % of the total attraction between the neutral units, but this includes the polarization within the fragments during bond formation. The dominance of covalent bonding remains when the final bonding situation is analyzed. Tables S4 and S5 show the EDA‐NOCV results for **5** and **6** using neutral and charged fragments as interacting moieties. The smallest Δ*E*_orb_ values are found when the singly charged species Be^+^ and (N_2_)_3_^−^ are used for the calculations, which is a measure for the best description of the bonds finally formed.^35^ The covalent (orbital) part of the attractive interactions still constitutes greater than 50 % of the total attraction.

The large Δ*E*_int_ values in Table 3 of **5** and **6** come from the interactions of the beryllium atom in the excited electron configuration (2s^0^2p^2^) with the ligands. We calculated the thermodynamic stabilities of the observed species **1**, **2**, **5**, and **6** for loss of the N_2_ ligands. The results are shown in Table 4. The most stable three‐coordinated complex **5** is thermodynamically stable, even at room temperature, for loss of one N_2_ yielding **1** by Δ*G* ^298^=9.0 kcal mol^−1^. The dissociation of all three N_2_ ligands from **5** and **6**, yielding a Be atom in the electronic ground‐state configuration is barely endergonic at 4 K by 4.4 kcal mol^−1^ and 2.5 kcal mol^−1^, respectively, but the reactions are exergonic at room temperature. The two‐coordinated complexes **1** and **2** even have negative bond dissociation energies and are thermodynamically unstable toward loss of the N_2_ ligands. The calculations suggest that **1** and **2** are only kinetically stable, consistent with the experimental observation that **1** and **2** are only formed on sample deposition during which some laser‐evaporated excited atoms are involved.

**Table 4:** Calculated bond dissociation energies *D*_e_ and reaction enthalpies at 4 K and 298 K at the CCSD(T)‐Full/cc‐pVTZ level. All the energy values are in kcal mol^−1^.

Reaction	*D*_e_	Δ*G* ^4^	Δ*G* ^298^
**5** → **1** + N_2_	20.0	18.0	9.0
**5** → Be + 3 N_2_	9.7	4.4	−18.9
**6** → Be + 3 N_2_	8.0	2.5	−20.9
**1** → Be + 2 N_2_	−10.2	−13.7	−27.9
**2** → Be + 2 N_2_	−11.8	−15.3	−28.8

The preference of formation of side‐on bonded dinitrogen complexes for the main‐group metal beryllium is quite unusual, as the end‐on coordination is the energetically preferred binding mode. All the experimentally known main‐group element complexes are end‐on bonded except the LiN_2_ complex, which was suggested to have a side‐on bonded structure as the bonding between Li and N_2_ is almost purely ionic.^20^ The end‐on mode is also energetically favored over the side‐on mode for mononuclear transition‐metal complexes, and the side‐on isomers could only be generated photochemically.^37^

## Conclusion

We report the preparation and spectroscopic identification of the complexes NNBe(η^2^‐N_2_) (**B**) and (NN)_2_Be(η^2^‐N_2_) (**C**) and the energetically higher lying isomers Be(NN)_2_ (**A**) and Be(NN)_3_ (**D**). The molecules **B** and **C** are the first examples of covalently side‐on bonded N_2_ adducts of a main‐group element. The analysis of the electronic structure using modern methods of quantum chemistry suggests that NNBe(η^2^‐N_2_) and (NN)_2_Be(η^2^‐N_2_) should be classified as π complexes rather than metalladiazirines. The energy preference of isomers with the side‐on bonded (η^2^‐N_2_) ligands is due to the large Be(2s^0^2p^2^)→η^2^‐N_2_ π back‐donation. Although the observed molecules are exotic and possess low thermodynamic stability, they enrich the arsenal of main‐group complexes with dinitrogen complexes with the first examples of side‐on bonded adducts. The information, which is gained by the bonding analysis, may be useful for the search of more stable dinitrogen complexes.

## Experimental Section

Experimental procedures and details about the theoretical work are given in the Supporting Information.

## Conflict of interest

The authors declare no conflict of interest.

## Supporting information

As a service to our authors and readers, this journal provides supporting information supplied by the authors. Such materials are peer reviewed and may be re‐organized for online delivery, but are not copy‐edited or typeset. Technical support issues arising from supporting information (other than missing files) should be addressed to the authors.

Supplementary
