# Supplementary material for: Side‐On Bonded Beryllium Dinitrogen Complexes
Source: Angew Chem Int Ed Engl. 2020 Apr 14;59(26):10603–9. doi: 10.1002/anie.202002621 (PMC7317369; doi:10.1002/anie.202002621)
Supplement: Supplementary file 1 — Supplementary [file ANIE-59-10603-s001.pdf]

Angewandte Chemie
Eine Zeitschrift der Gesellschaft Deutscher Chemiker

Supporting Information

Side-On Bonded Beryllium Dinitrogen Complexes

Guohai Deng+, Sudip Pan+, Guanjun Wang, Lili Zhao, Mingfei Zhou, and Gernot Frenking**

anie_202002621_sm_miscellaneous_information.pdf

Supporting Information

1. Experimental and computational details
2. Figures S1 – S7
3. Tables S1 – S3

Experimental and computational details

The 1064 nm fundamental of a Nd:YAG laser (Continuum, Minilite II, 10 Hz repetition rate and 6 ns pulse width) was used to produce beryllium atoms. The laser beam was focused onto a rotating beryllium metal target through a hole in a CsI window at 4 K. The laser-evaporated beryllium atoms were co-deposited with N_2/Ne mixtures or pure dinitrogen onto the CsI window. In general, matrix samples were deposited for 30 minutes at a rate of 4-8 mmol/h. The N_2/Ne mixtures were prepared in a stainless steel vacuum line using standard manometric technique. After sample deposition, infrared spectra of the resulting samples were recorded on a Bruker V80 spectrometer at a 0.5 cm^{-1} resolution between 4000 and 450 cm^{-1} using a liquid nitrogen cooled broad band HgCdTe (MCT) detector. Bare mirror backgrounds, recorded prior to sample deposition were used as references in processing the sample spectra. The spectra were subjected to baseline correction to compensate for infrared light scattering and interference patterns. Samples were annealed to different temperatures and cooled back to 4 K for spectral acquisition. For selected samples, photo-excitations were performed through a quartz window mounted on the assembly.

The geometries and vibrational spectra of $Be(NN)_n$ **1 – 8** and N_2 were optimized at the CCSD(T)-Full/cc-pVTZ level.[1] Further single-point energy calculations on the optimized structures of **1**, **2**, **5** and **6** were carried out with larger basis sets at the CCSD(T)-Full/aug-cc-pVQZ[1d,1e] level using the CCSD(T)-Full/cc-pVTZ optimized geometries. All structures are minima on the potential energy surface. All these calculations were carried out with Gaussian 16.[2] The NBO calculations were carried out with the version 6.0.[3] The QTAIM (Quantum Theory of Atoms in Molecules) analyses were performed with the AIMALL program[4] using the wave function at the CCSD-Full/cc-pVTZ level.

The bonding situations of **1, 2, 5** and **6** were further analyzed by means of an energy decomposition analysis (EDA)[5] together with the natural orbitals for chemical valence (NOCV)[6] method by using the ADF 2018.105 program package.[7] The EDA-NOCV calculations[8] were carried out at the M06-2X/TZ2P level using the

CCSD(T)-Full/cc-pVTZ optimized geometries. TZ2P is a triple-ζ quality basis set augmented by two sets of polarization functions.[9] In this analysis, the intrinsic interaction energy (ΔE_{int}) between two fragments can be divided into three energy components as follows:

$$\Delta E_{\text{int}} = \Delta E_{\text{elstat}} + \Delta E_{\text{Pauli}} + \Delta E_{\text{orb}} \qquad (1).$$

The electrostatic ΔE_{elstat} term represents the quasiclassical electrostatic interaction between the unperturbed charge distributions of the prepared fragments, the Pauli repulsion ΔE_{Pauli} corresponds to the energy change associated with the transformation from the superposition of the unperturbed electron densities of the isolated fragments to the wavefunction, which properly obeys the Pauli principle through explicit antisymmetrization and renormalization of the production wavefunction. The orbital term ΔE_{orb} is originated from the mixing of orbitals, charge transfer and polarization between the isolated fragments, which can be further decomposed into contributions from each irreducible representation of the point group of the interacting system as follows:

$$\Delta E_{orb} = \sum_{r} \Delta E_{r} \qquad (2)$$

The combination of the EDA with NOCV[9] enables the partition of the total orbital interactions into pairwise contributions of the orbital interactions which is very vital to get a complete picture of the bonding. The charge deformation $\Delta\rho_k(r)$, resulting from the mixing of the orbital pairs $\psi_{\text{k}}(\text{r})$ and $\psi_{\text{-k}}(\text{r})$ of the interacting fragments presents the amount and the shape of the charge flow due to the orbital interactions (Equation 3), and the associated energy term ΔE_{orb} provides with the size of stabilizing orbital energy originated from such interaction (Equation 4).

$$\Delta\rho_{orb}(r) = \sum_{k} \Delta\rho_k(r) = \sum_{k=1}^{N/2} \nu_k [-\psi_{-k}^2(r) + \psi_k^2(r)] \qquad (3)$$

$$\Delta E_{orb} = \sum_{k} \Delta E_k^{orb} = \sum_{k=1}^{N/2} \nu_k [-F_{-k,-k}^{TS} + F_{k,k}^{TS}] \qquad (4)$$

Since we used a metahybrid functional, which has energy contributions that cannot be assigned to the three EDA terms, the calculations give a physically unspecified addition

terms $\Delta E_{\mathrm{Metahybrid,}}$ which is rather small. More details about the EDA-NOCV method and its application are given in recent reviews articles[10].

References

[1] a) G. D. Purvis III, R. J. Bartlett, *J. Chem. Phys*. **1982**, *76*, 1910; b) J. A. Pople, M. Head-Gordon, K. Raghavachari, *J. Chem. Phys*. **1987**, *87*, 5968; c) T. H. Dunning Jr., *J. Chem. Phys*. **1989**, *90*, 1007; d) R. A. Kendall, T. H. Dunning Jr., R. J. Harrison, *J. Chem. Phys*. **1992**, *96*, 6796; e) D. E. Woon, T. H. Dunning Jr., *J. Chem. Phys*. **1993**, *98*, 1358; f) K. A. Peterson, D. E. Woon, T. H. Dunning Jr., *J. Chem. Phys*. **1994**, *100*, 7410.

[2] Gaussian 16, Revision A.03, M. J. Frisch, G. W. Trucks, H. B. Schlegel, G. E. Scuseria, M. A. Robb, J. R. Cheeseman, G. Scalmani, V. Barone, G. A. Petersson, H. Nakatsuji, X. Li, M. Caricato, A. V. Marenich, J. Bloino, B. G. Janesko, R. Gomperts, B. Mennucci, H. P. Hratchian, J. V. Ortiz, A. F. Izmaylov, J. L. Sonnenberg, D. Williams-Young, F. Ding, F. Lipparini, F. Egidi, J. Goings, B. Peng, A. Petrone, T. Henderson, D. Ranasinghe, V. G. Zakrzewski, J. Gao, N. Rega, G. Zheng, W. Liang, M. Hada, M. Ehara, K. Toyota, R. Fukuda, J. Hasegawa, M. Ishida, T. Nakajima, Y. Honda, O. Kitao, H. Nakai, T. Vreven, K. Throssell, J. A. Montgomery, Jr., J. E. Peralta, F. Ogliaro, M. J. Bearpark, J. J. Heyd, E. N. Brothers, K. N. Kudin, V. N. Staroverov, T. A. Keith, R. Kobayashi, J. Normand, K. Raghavachari, A. P. Rendell, J. C. Burant, S. S. Iyengar, J. Tomasi, M. Cossi, J. M. Millam, M. Klene, C. Adamo, R. Cammi, J. W. Ochterski, R. L. Martin, K. Morokuma, O. Farkas, J. B. Foresman, and D. J. Fox, Gaussian, Inc., Wallingford CT, **2016**.

[3] E. D. Glendening, C. R. Landis, C.F. Weinhold, F. *J. Comput. Chem*. **2013,** *34*, 1429.

[4] AIMAll (Version 17.11.14), T. A. Keith, TK Gristmill Software, Overland Park KS, USA, **2017.**

[5] T. Ziegler, A. Rauk, *Theor. Chim. Acta* **1977**, *46*, 1-10.

[6] a) M. Mitoraj, A. Michalak, *Organometallics* **2007**, *26*, 6576; b) M. Mitoraj, A. Michalak, *J. Mol. Model.* **2008**, *14*, 681.

[7] a) ADF2018, SCM, Theoretical Chemistry, Vrije Universiteit, Amsterdam, The Netherlands, http://www.scm.com; b) G. te Velde, F. M. Bickelhaupt, E. J. Baerends, C. F. Guerra, S. J. A. Van Gisbergen, J. G. Snijders, T. Ziegler, *J. Comput. Chem*. **2001**, *22*, 931.

[8] a) A. Michalak, M. Mitoraj, T. Ziegler, *J. Phys. Chem. A* **2008,** *112*, 1933; b) M. P. Mitoraj, A. Michalak, T. Ziegler, *J. Chem. Theory Comput.* **2009**, *5*, 962.
[9] E. van Lenthe, E. J. Baerends, *J. Comput. Chem*. **2003**, *24*, 1142.
[10] a) L. Zhao, M. von Hopffgarten, D. M. Andrada, G. Frenking, *WIREs Comput. Mol. Sci.*, **2018**, *8*, e1345; b) G. Frenking, F. M. Bickelhaupt, in *The Chemical Bond. Fundamental Aspects of Chemical Bonding*, G. Frenking and S. Shaik (Eds), Wiley-VCH, Weinheim, **2014**, p. 121-158; c) G. Frenking, R. Tonner, S. Klein, N. Takagi, T. Shimizu, A. Krapp, K. K. Pandey, P. Parameswaran, *Chem. Soc. Rev*. **2014**, *43*, 5106; e) L. Zhao, M. Hermann, N. Holzmann, G. Frenking, *Coord. Chem. Rev*. **2017**, *344*, 163; f) G. Frenking, M. Hermann, D. M. Andrada, N. Holzmann, *Chem. Soc. Rev*. **2016**, *45*, 1129; g) L. Zhao, M. Hermann, W.H.E. Schwarz, G. Frenking, *Nat. Rev. Chem.* **2019**, *3*, 48; h) L. Zhao, S. Pan, N. Holzmann, P. Schwerdtfeger, G. Frenking, *Chem. Rev*. **2019**, *119*, 8781.

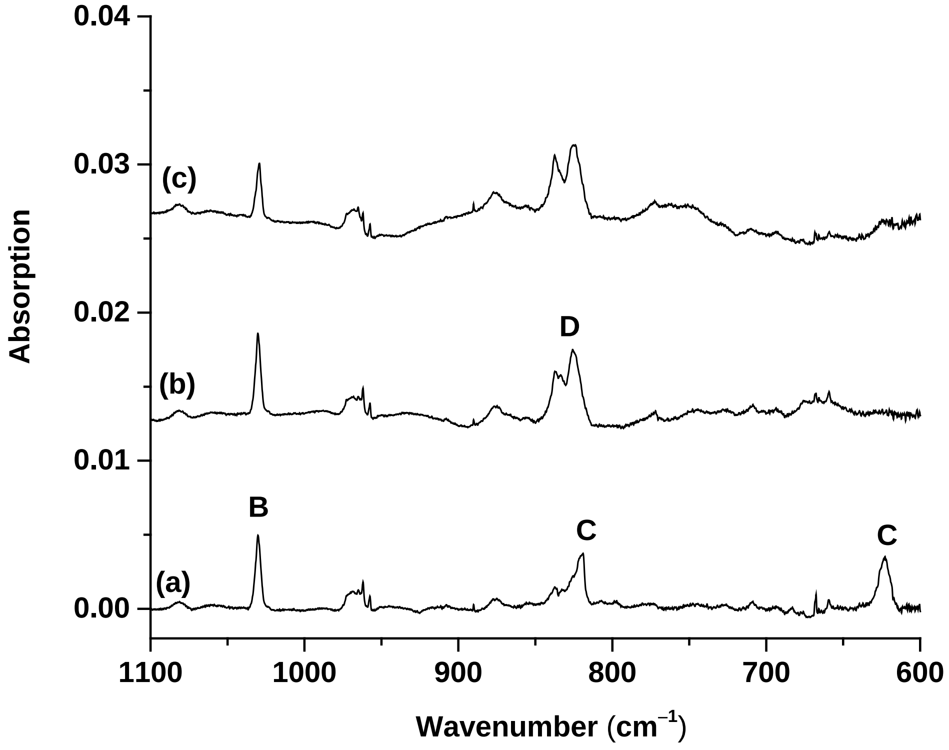

Figure S1. Infrared spectra in the 1100–600 cm^{-1} region from co-deposition of laser-evaporated beryllium atoms with 0.5% N_2 in neon. (a) 30 min of sample deposition at 4 K; (b) after 4 min of 617 nm light irradiation; and (c) after annealing to 10 K. **B**: NNBe(η^2-N_2); **C**: $(NN)_2$Be(η^2-N_2); **D**: $Be(NN)_3$.

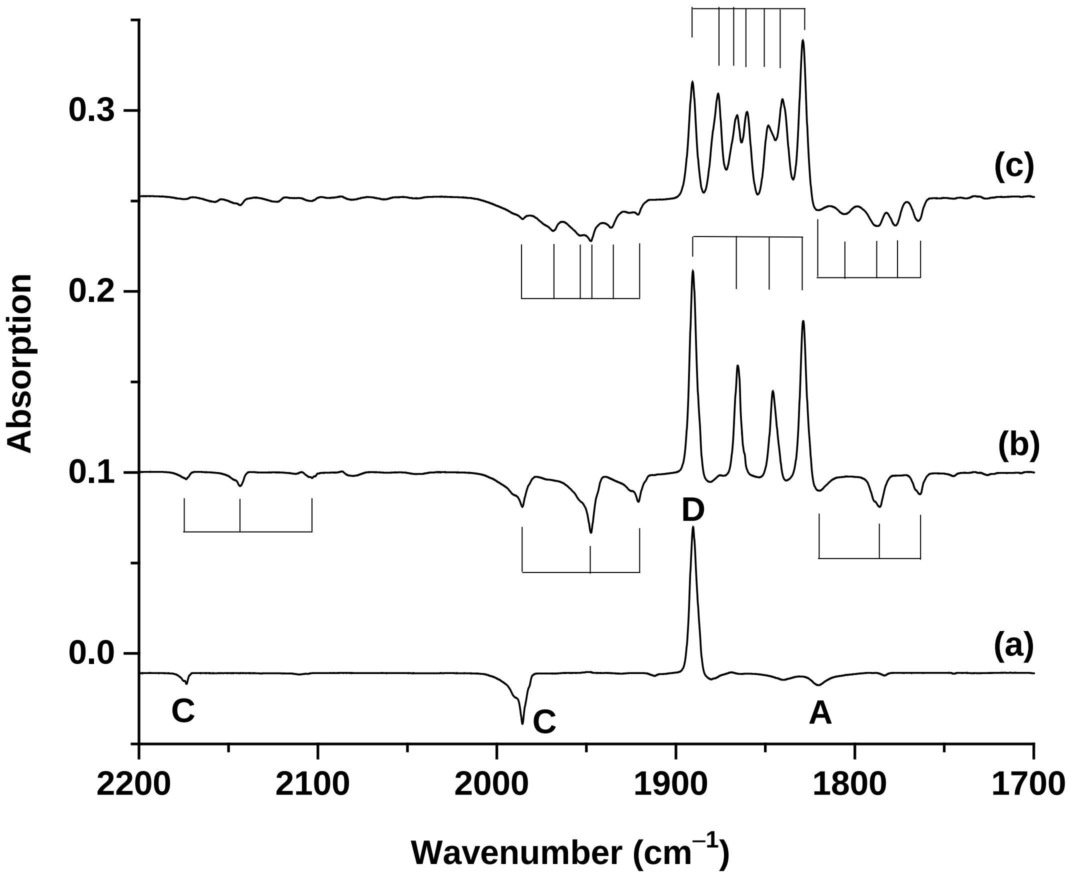

Figure S2. Difference infrared spectra in the 2200–1700 cm^{-1} region from co-deposition of laser-evaporated beryllium atoms with isotope-labeled N_2 in excess neon. Spectra were taken after 4 min of 617 nm light irradiation. (a) 0.5% $^{14}N_2$; (b) 0.5% $^{14}N_2$ + 0.5% $^{15}N_2$, and (c) 0.25% $^{14}N_2$ + 0.5% $^{14}N^{15}N$ + 0.25% $^{15}N_2$. **A**: Be(NN)$_2$; **C**: (NN)$_2$Be(η^2-N$_2$); **D**:Be(NN)$_3$.

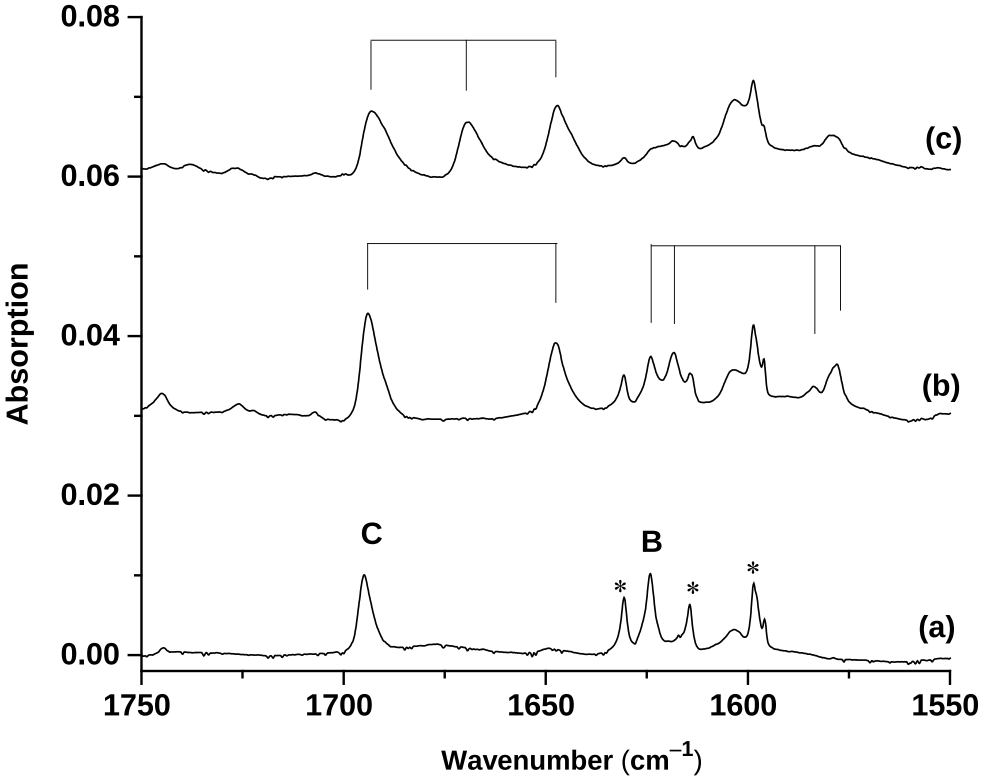

Figure S3. Difference infrared spectra in the 1750–1550 cm^{-1} region from co-deposition of laser-evaporated beryllium atoms with isotope-labeled N_2 in excess neon. Spectra were taken after sample deposition at 4 K. (a) 0.5% $^{14}N_2$; (b) 0.5% $^{14}N_2$ + 0.5% $^{15}N_2$, and (c) 0.25% $^{14}N_2$ + 0.5% $^{14}N^{15}N$ + 0.25% $^{15}N_2$. **B**: NNBe(η^2-N_2); **C**: $(NN)_2$Be(η^2-N_2). The * denotes water absorptions.

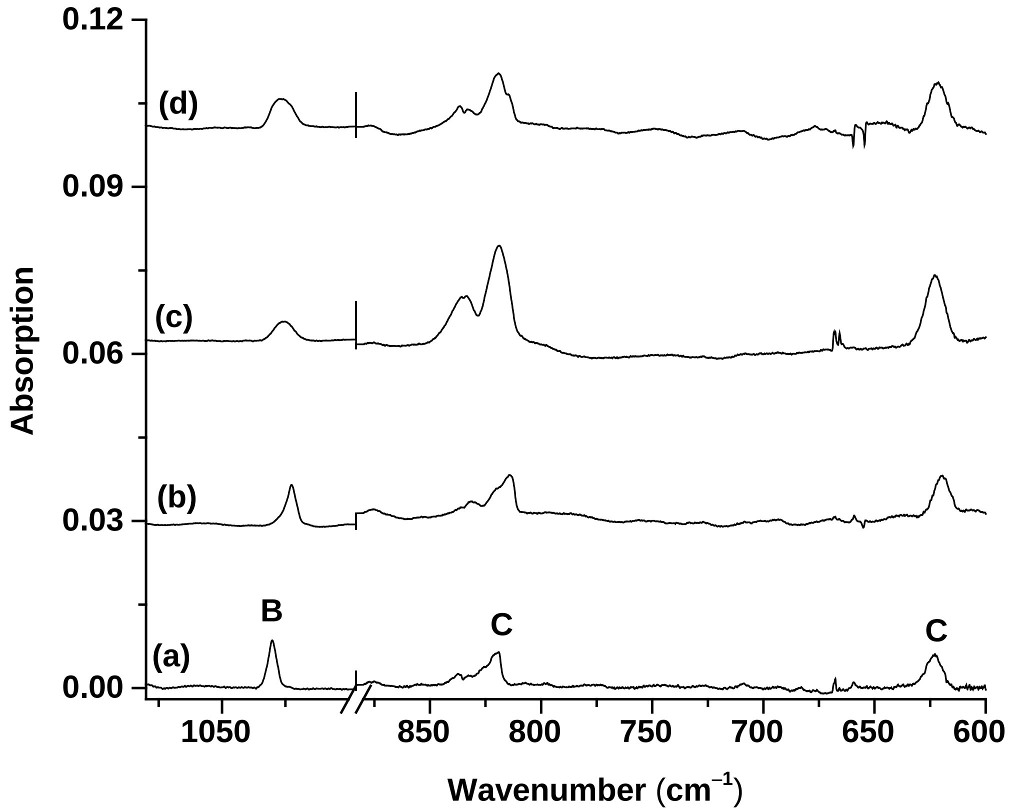

Figure S4. Infrared spectra in the 1080-1000 and 880–600 cm^{-1} regions from co-deposition of laser-evaporated beryllium atoms with isotope-labeled N_2 in excess neon. Spectra were taken after sample deposition at 4 K. (a) 0.5% $^{14}N_2$; (b) 0.5% $^{15}N_2$; (c) 0.5% $^{14}N_2$ + 0.5% $^{15}N_2$, and (d) 0.25% $^{14}N_2$ + 0.5% $^{14}N^{15}N$ + 0.25% $^{15}N_2$. **B**: NNBe(η^2-N_2); **C**: $(NN)_2$Be(η^2-N_2).

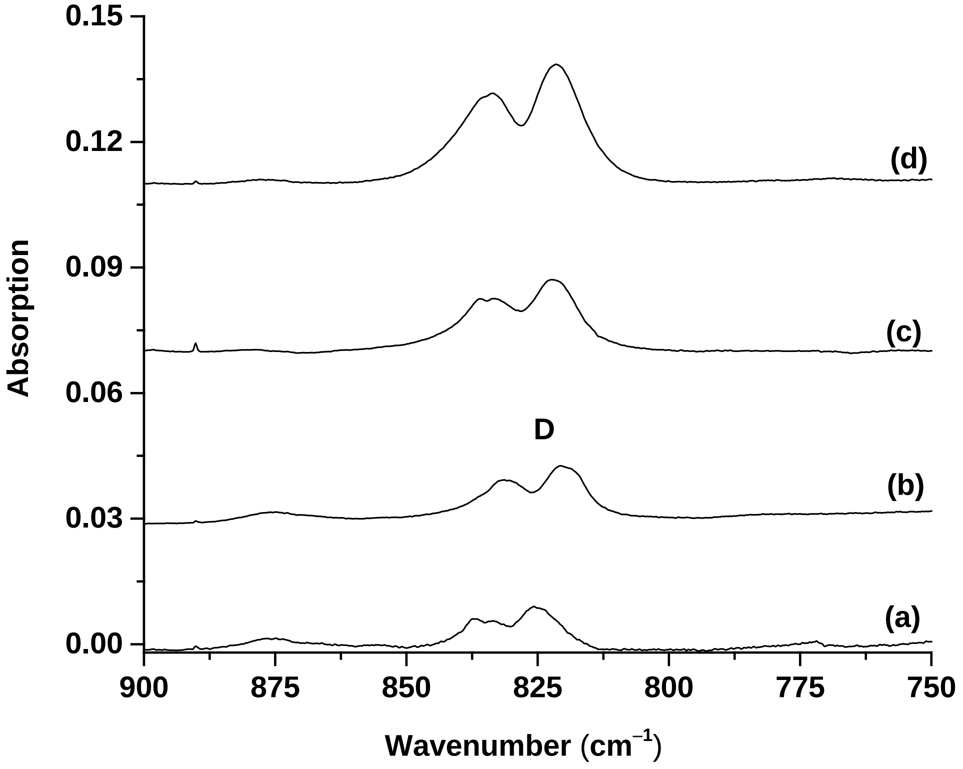

Figure S5. Infrared spectra in the 900-750 cm^{-1} region from co-deposition of laser-evaporated beryllium atoms with isotope-labeled N_2 in excess neon. Spectra were taken after sample deposition at 4 K followed by 4 min of 617 nm light irradiation. (a) 0.5% $^{14}N_2$; (b) 0.5% $^{15}N_2$; (c) 0.5% $^{14}N_2$ + 0.5% $^{15}N_2$, and (d) 0.25% $^{14}N_2$ + 0.5% $^{14}N^{15}N$ + 0.25% $^{15}N_2$. **D**: Be(NN)$_3$.

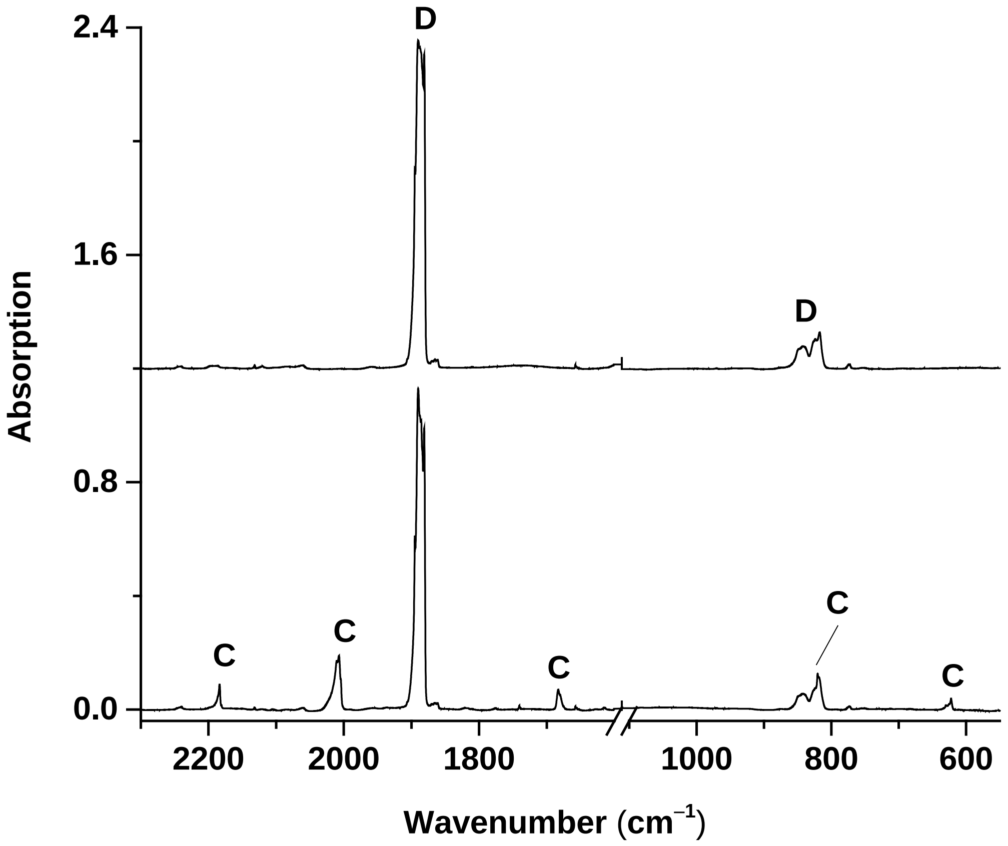

Figure S6. Infrared spectra in the 2300–1600 and 1100–550 cm^{-1} regions from co-deposition of laser-evaporated beryllium atoms with N_2. (a) 40 min of sample deposition at 4 K; and (b) after 4 min of 617 nm light irradiation. **C**: $(NN)_2Be(\eta^2\text{-}N_2)$; **D**: $Be(NN)_3$.

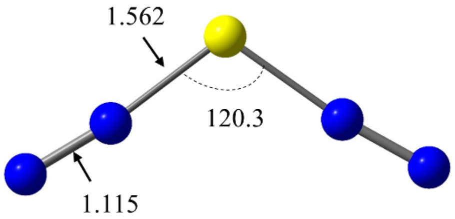

2, E_{rel}: 0.0

Be(NN)$_2$ (C_{2v}, $^3B_1$)

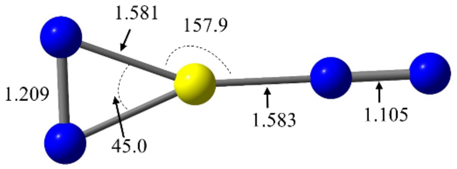

1, E_{rel}: 5.9

Be(NN)(η^2-N$_2$) (C_{2v}, $^1A_1$)

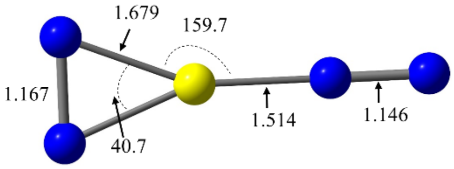

3, E_{rel}: 5.4

Be(NN)(η^2-N$_2$) (C_{2v}, $^3A_2$)

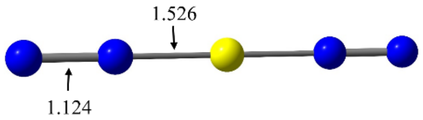

4, E_{rel}: 19.1

Be(NN)$_2$ ($D_{\infty h}$, $^1\Sigma_g$)

(NIMAG = 1, 88.2i)

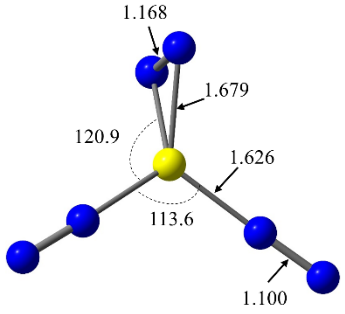

5, E_{rel}: 0.0

Be(NN)$_2$(η^2-N$_2$) (C_{2v}, $^1A_1$)

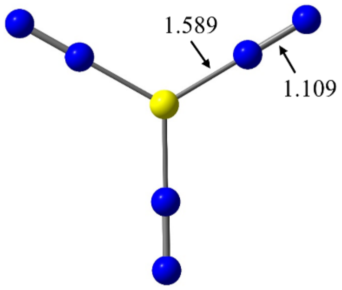

6, E_{rel}: 0.3

Be(NN)$_3$ (D_{3h}, $^1A_1'$)

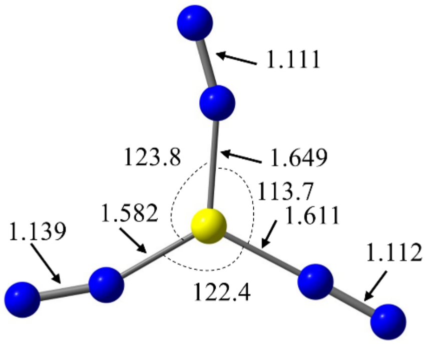

7, E_{rel}: 15.4

Be(NN)$_3$ (C_s, $^3A'$)

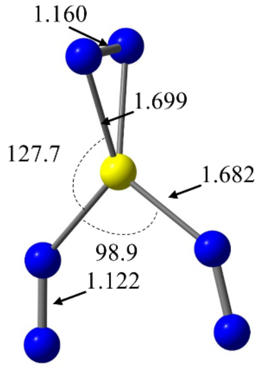

8, E_{rel}: 16.6

Be(NN)$_2$(η^2-N$_2$) (C_{2v}, $^3B_1$)

Figure S7. Minimum energy geometries of di- and tri-coordinated beryllium-dinitrogen complexes at the M06-2X-D3/cc-pVTZ level. Relative energies are given in kcal/mol. Bond distances are in Å and bond angles are in degree.

Table S1. Infrared absorptions (in cm^{-1}) observed from co-deposition of laser-evaporated beryllium atoms with dinitrogen in solid neon at 4 K.

Species	$^{14}N_2$	$^{15}N_2$	$^{14}N_2$ + $^{15}N_2$	$^{14}N_2$ + $^{14}N^{15}N$ + $^{15}N_2$
$Be(NN)_2$ (**A**)	1820.4	1763.5	1819.8, 1786.0, 1763.5	1820.4, 1805.9, 1787.4, 1777.2, 1764.6
NNBe(η^2-N_2) (**B**)	1948.9	1887.6		
	1624.1	1577.8	1624.0, 1618.2,1583.7, 1577.8	1618.3, 1603.1, 1579.1
	1030.2	1022.5	1025.2	1025.9
$(NN)_2$Be(η^2-N_2) (**C**)	2173.5	2101.5	2173.5, 2143.3, 2103.3	2174.1, 2157.7, 2143.3, 2122.9, 2103.9,
	1985.8	1920.7	1985.8, 1947.5, 1920.7	1985.7, 1968.6, 1953.5, 1947.5, 1936.2, 1921.1
	1694.9	1647.0	1693.9, 1647.4	1692.9, 1669.2, 1647.0
	819.0	813.3		
	622.8	619.5	622.5	621.3
$Be(NN)_3$ (**D**)	1890.3	1828.8	1890.5, 1865.4, 1845.9, 1828.9	1890.8, 1876.3, 1865.9, 1860.2, 1848.1, 1840.5, 1828.8
	836.5/825.0	831.1/819.8		

Table S2. The unscaled vibrational frequencies of the calculated molecules at the CCSD(T)-Full/cc-pVTZ level.

Molecule	ν (cm^{-1})
$Be(N_2)(\eta^2\text{-}N_2)$ (C_{2v}, $^1A_1$)	108.6
	147.5
	385.2
	412.2
	468.8
	826.7
	1088.7
	1647.3
	2034.2
$Be(N_2)_2$ (C_{2v}, $^3B_1$)	103.0
	322.8
	360.7
	367.6
	419.4
	599.8
	788.6
	1972.5
	2171.0
$Be(N_2)(\eta^2\text{-}N_2)$ (C_s, $^3A''$)	137.3
	172.3
	262.0
	364.6
	473.8
	536.9
	1222.8

	1823.2<br>2031.3
$Be(N_2)_2$ ($D_{\infty h}$, $^1\Sigma_g$)	62.5<br>82.1<br>324.7<br>390.8<br>417.1<br>438.1<br>483.5<br>1284.1<br>1927.9<br>1976.1
$Be(N_2)_2(\eta^2\text{-}N_2)$ (C_{2v}, $^1A_1$)	80.4<br>117.9<br>138.3<br>186.3<br>320.7<br>370.0<br>407.6<br>438.9<br>451.6<br>683.0<br>759.3<br>861.4<br>1715.0<br>2077.3<br>2241.1
$Be(N_2)_3$ (D_{3h}, $^1A_1'$)	61.8<br>73.9<br>109.8

	294.8
	357.1
	362.2
	407.5
	407.5
	423.6
	505.5
	891.4
	891.4
	1971.7
	1972.3
	2190.4

Table S3. The unscaled vibrational frequencies and intensities of the calculated molecules at the M06-2X-D3/cc-pVTZ level.

Molecule	ν (cm^{-1})	*I* (km/mol)
Be(NN)(η^2-N_2) (C_{2v}, $^1A_1$)	96.5	2.1
	116.1	36.7
	357.9	17.3
	436.8	6.7
	458.9	0.1
	954.7	7.5
	1032.9	926.4
	1723.8	673.8
	2313.3	862.6
$Be(NN)_2$ (C_{2v}, $^3B_1$)	94.2	0.1
	356.4	2.3
	377.5	0.0
	389.7	9.5

	398.4	7.2
	616.3	46.1
	949.8	556.3
	1997.3	3741.8
	2220.6	481.2
Be(NN)(η^2-N_2) (C_{2v}, $^3A_2$)	122.9	15.9
	137.4	14.2
	277.3	0.0
	363.5	5.1
	471.5	1.8
	555.9	11.5
	1209.7	151.3
	1957.1	92.8
	2066.1	110.4
Be(NN)$_2$ ($D_{\infty h}$, $^1\Sigma_g$)	-88.2	6.2
	73.2	19.2
	306.9	0.0
	398.6	26.2
	453.9	0.0
	477.1	55.2
	478.7	0.0
	1222.1	351.9
	2059.0	3598.7
	2203.065	0
Be(NN)$_2$(η^2-N_2) (C_{2v}, $^1A_1$)	95.0	0.3
	129.7	0.1
	138.0	8.1
	198.7	0.0
	333.1	4.0
	379.4	1.5

	416.6	0.1
	461.0	9.4
	467.3	0.0
	785.7	2.6
	787.9	63.7
	799.0	399.2
	1875.5	1045.5
	2292.6	1296.8
	2384.4	329.1
$Be(NN)_3$ (D_{3h}, $^1A_1'$)	95.2	1.6
	95.2	1.6
	101.5	1.3
	307.8	0.0
	377.1	3.1
	377.1	3.1
	419.5	0.0
	419.5	0.0
	425.8	0.0
	533.4	21.4
	926.2	131.1
	926.5	131.1
	2201.3	2098.1
	2201.3	2098.2
	2350.1	0.0
$Be(NN)_3$ (C_s, $^3A'$)	49.6	74.3
	87.9	15.7
	113.4	4.7
	223.2	32.4
	227.0	0.2
	275.2	415.8

	355.2	132.0
	364.3	0.1
	396.0	0.0
	404.9	68.8
	586.7	1883.5
	836.9	758.0
	1577.4	11775.2
	2095.1	2574.8
	2288.3	312.6
$Be(NN)_2(\eta^2\text{-}N_2)$ (C_{2v}, $^3B_1$)	123.9	9.8
	126.9	0.1
	166.1	3.1
	170.3	0.0
	295.4	0.0
	343.0	6.1
	350.3	5.4
	370.5	128.9
	431.1	0.9
	511.9	135.8
	557.6	12.2
	944.2	45.6
	1792.0	5152.4
	1981.1	289.8
	2285.5	5.5

Table S4. Coordinates and energies of the calculated molecules at the CCSD(T)-Full/cc-pVTZ level.

$Be(N_2)(\eta^2\text{-}N_2)$ (C_{2v}, $^1A_1$)<br>E = -233.4072717 au

Be	0.000000000	0.000000000	-0.270845000
N	-0.000000000	-0.000000000	1.275379000
N	-0.000000000	-0.000000000	2.406431000
N	0.000000000	0.607302000	-1.763521000
N	-0.000000000	-0.607302000	-1.763521000
Be$(N_2)_2$ (C_{2v}, $^3B_1$)			
E = -233.4048007 au			
Be	-0.000000000	0.000000000	1.056828000
N	0.000000000	1.300892000	0.125307000
N	0.000000000	2.275276000	-0.427258000
N	-0.000000000	-1.300892000	0.125307000
N	-0.000000000	-2.275276000	-0.427258000
Be$(N_2)(\eta^2$-$N_2)$ (C_s, $^3A''$)			
E = -233.3947477			
Be	-0.000000000	0.230097000	-0.000000000
N	-0.599962000	1.792109000	-0.000000000
N	0.586410000	1.797550000	0.000000000
N	0.004911000	-1.280813000	0.000000000
N	0.008641000	-2.440331000	0.000000000
Be$(N_2)_2$ ($D_{\infty h}$, $^1\Sigma_g$)			
E = -233.3917676			
N	0.000000000	0.000000000	2.660810000
N	0.000000000	0.000000000	1.511976000
Be	0.000000000	0.000000000	0.000000000
N	0.000000000	0.000000000	-1.511976000
N	0.000000000	0.000000000	-2.660810000
Be$(N_2)_2(\eta^2$-$N_2)$ (C_{2v}, $^1A_1$)			
E = -342.8389821 au			

Be	0.000000000	0.000000000	0.268936000
N	-0.596966000	0.000000000	1.828367000
N	0.596966000	-0.000000000	1.828367000
N	0.000000000	1.340680000	-0.653825000
N	-0.000000000	-1.340680000	-0.653825000
N	0.000000000	2.279792000	-1.251381000
N	-0.000000000	-2.279792000	-1.251381000
$Be(N_2)_3$ (D_{3h}, $^1A_1'$)			
E = -342.8362521 au			
Be	0.000000000	0.000000000	0.000000000
N	0.000000000	1.579829000	0.000000000
N	0.000000000	2.703956000	0.000000000
N	1.368172000	-0.789914000	0.000000000
N	2.341694000	-1.351978000	0.000000000
N	-1.368172000	-0.789914000	0.000000000
N	-2.341694000	-1.351978000	0.000000000

Table S5. Coordinates and energies of the calculated molecules at the M06-2X-D3/cc-pVTZ level.

$Be(N_2)(\eta^2-N_2)$ (C_{2v}, $^1A_1$)			
E = -233.7214976 au			
Be	0.000000000	0.000000000	-0.295068000
N	0.000000000	-0.000000000	1.287606000
N	0.000000000	-0.000000000	2.392290000
N	-0.000000000	0.604634000	-1.755643000
N	-0.000000000	-0.604634000	-1.755643000
$Be(N_2)_2$ (C_{2v}, $^3B_1$)			
E = -233.7309376 au			
Be	0.000000000	-0.000000000	0.887809000

N	-0.000000000	1.354642000	0.110673000
N	-0.000000000	2.363578000	-0.364333000
N	-0.000000000	-1.354642000	0.110673000
N	-0.000000000	-2.363578000	-0.364333000
$Be(N_2)(\eta^2\text{-}N_2)$ (C_{2v}, $^3A_2$)			
E = -233.72233 au			
Be	0.000000000	-0.000000000	-0.224530000
N	0.000000000	0.583651000	-1.798610000
N	-0.000000000	-0.583651000	-1.798610000
N	0.000000000	0.000000000	1.289903000
N	0.000000000	0.000000000	2.435618000
$Be(N_2)_2$ ($D_{\infty h}$, $^1\Sigma_g$)			
E = -233.70043 au			
Be	0.000000000	0.000000000	0.000000000
N	0.000000000	0.000000000	1.525793000
N	0.000000000	0.000000000	-1.525793000
N	0.000000000	0.000000000	-2.649603000
N	-0.000000000	0.000000000	2.649603000
$Be(N_2)_2(\eta^2\text{-}N_2)$ (C_{2v}, $^1A_1$)			
E = -343.29121 au			
Be	0.000000000	0.000000000	0.237948000
N	-0.583848000	0.000000000	1.812617000
N	0.583848000	-0.000000000	1.812617000
N	0.000000000	1.360534000	-0.653079000
N	-0.000000000	-1.360534000	-0.653079000
N	0.000000000	2.299190000	-1.227523000
N	-0.000000000	-2.299190000	-1.227523000
$Be(N_2)_3$ (D_{3h}, $^1A_1'$)			
E = -343.29078 au			
Be	0.000000000	0.000000000	0.000000000

N	0.000000000	1.588555000	0.000000000
N	0.000000000	2.697452000	0.000000000
N	1.375729000	-0.794277000	0.000000000
N	2.336062000	-1.348726000	0.000000000
N	-1.375729000	-0.794277000	0.000000000
N	-2.336062000	-1.348726000	0.00000000
$Be(N_2)_2(\eta^2\text{-}N_2)$ (C_{2v}, $^3B_1$)			
E = -343.2647031 au			
Be	0.000000000	0.000000000	0.517316000
N	-0.579983000	0.000000000	2.114538000
N	0.579983000	-0.000000000	2.114538000
N	0.000000000	1.278029000	-0.575476000
N	-0.000000000	-1.278029000	-0.575476000
N	0.000000000	1.431675000	-1.686866000
N	-0.000000000	-1.431675000	-1.686866000
$Be(N_2)_3$ (C_s, $^3A''$)			
E = -343.2666836 au			
Be	0.000000000	0.085925000	-0.000000000
N	-1.498528000	0.591630000	0.000000000
N	-2.637387000	0.565467000	0.000000000
N	0.432018000	-1.505219000	-0.000000000
N	0.347733000	-2.613409000	0.000000000
N	1.253549000	1.097529000	-0.000000000
N	2.102615000	1.814902000	-0.000000000
